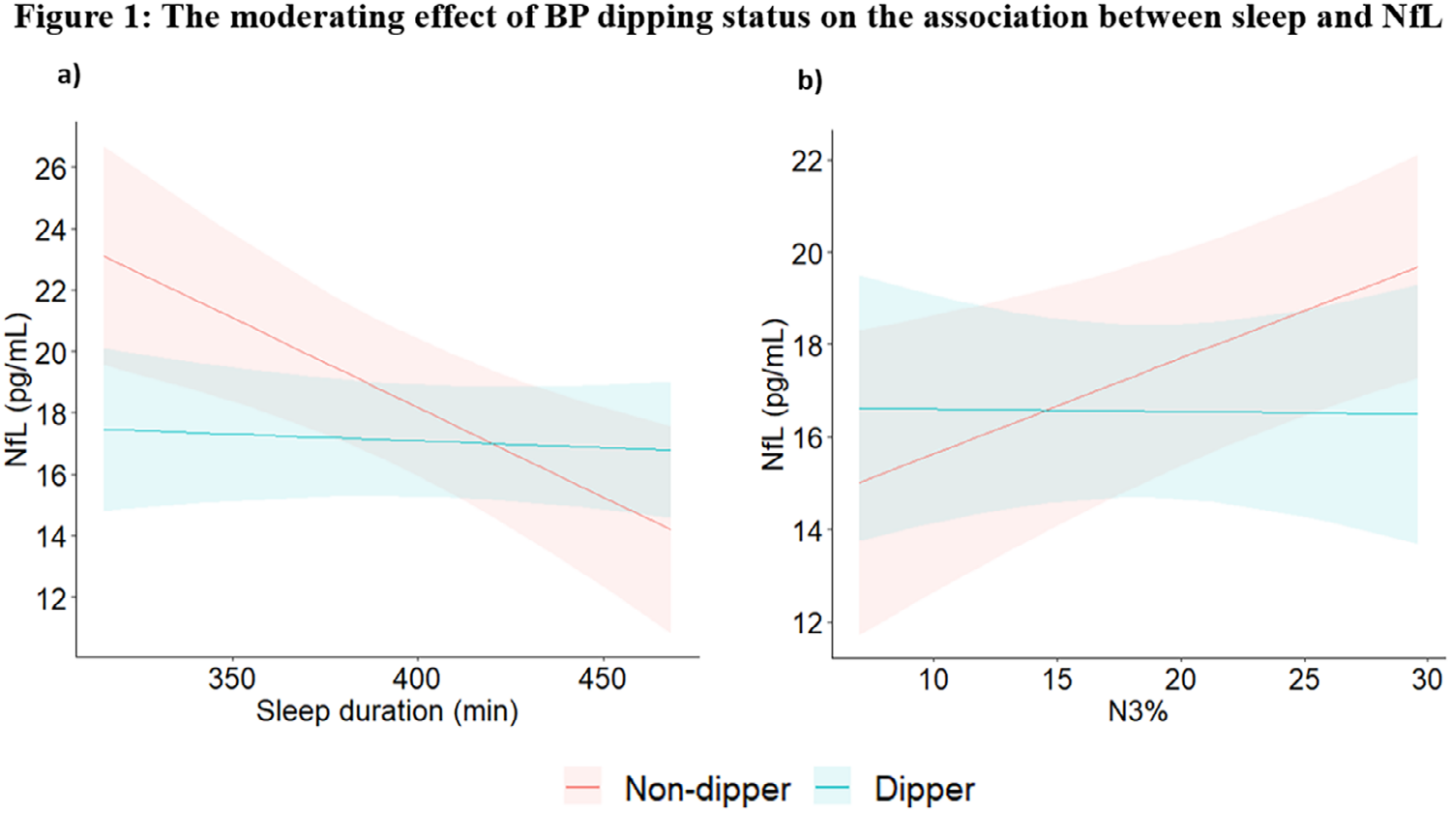# Short sleep and nocturnal hypertension: A combined risk for neuronal damage

**DOI:** 10.1002/alz70860_105351

**Published:** 2025-12-23

**Authors:** Stephanie Yiallourou, Madeline Gibson, Emma Louise Palatsides, Stuart McDonald, William O'Brien, Ming Ann Sim, Marina G. Cavuoto, Jayandra Jung Himali, Terence J O'Brien, Meng Law, Trevor T.‐J. Chong, Ian H Harding, Lucy Vivash, Matthew T Naughton, Garun S Hamilton, Matthew P. Pase

**Affiliations:** ^1^ School of Psychological Sciences & Turner Institute for Brain and Mental Health, Monash University, Melbourne, VIC, Australia; ^2^ Monash University, Clayton, VIC, Australia; ^3^ School of Psychological Sciences & Turner Institute for Brain and Mental Health, Monash University, Clayton, VIC, Australia; ^4^ Monash University, Melbourne, VIC, Australia; ^5^ Turner Institute for Brain and Mental Health & School of Psychological Sciences, Monash University, Clayton, VIC, Australia; ^6^ National University Hospital, Singapore, Singapore, Singapore; ^7^ National Ageing Research Institute, Melbourne, VIC, Australia; ^8^ University of Texas Health San Antonio, San Antonio, TX, USA; ^9^ Alfred Health, Melbourne, VIC, Australia; ^10^ Central Clinical School, Monash University, Melbourne, VIC, Australia; ^11^ Department of Respiratory Medicine, Alfred Health and Central Clinical, Melbourne, VIC, Australia; ^12^ Monash Lung, Sleep, Allergy and Immunology, Monash Health, Clayton, VIC, Australia

## Abstract

**Background:**

The relationship between short sleep duration and increased Alzheimer's disease (AD) risk remains equivocal. Disturbed sleep can lead to nocturnal hypertension, where the normal blood pressure (BP) dip during sleep (a dip in BP≥10% from daytime levels) is abolished, or in severe cases, reversed. Since high BP is a dementia risk factor, short sleep may increase risk of AD pathology via nocturnal hypertension. We assessed whether the association of sleep duration with AD plasma biomarkers was moderated by BP dipping status.

**Method:**

Dementia‐free participants aged ≥55 years underwent home‐based polysomnography, 24‐hour ambulatory BP monitoring and blood collection. SIMOA‐measured^®^ plasma *p*‐Tau217, GFAP, Aβ42/Aβ40 ratio, and NfL were obtained. Participants were categorized as “BP Dippers” (≥10% nocturnal dip in BP) and “Non‐Dippers” (<10% nocturnal dip in BP, reflecting nocturnal hypertension). Linear regressions were performed between sleep duration (min) and plasma biomarkers, adjusted for demographic factors, BMI, diabetic status, anti‐depressant and hypertensive medication use. An interaction term for BP dipping status was included in regression models. For those models yielding significant interactions, we performed an exploratory moderation analysis investigating sleep stages (N1, N2, N3, REM%) x BP dipping interactions.

**Result:**

In total, 92 participants were studied (mean age 67±5 years; 64% female); 35% were Non‐Dippers. BP dipping status moderated the association between sleep duration and NfL levels. Shorter sleep duration was associated with higher levels of NfL in Non‐Dippers (β = ‐0.06 [95%CI, ‐0.09, ‐0.02] per pg/mL, *p* = 0.001), but not in BP dippers (β = ‐0.004 [95%CI, ‐0.03, 0.02, *p* = 0.691) (Figure 1a). When sleep stages were explored, dipping status moderated the association between slow wave sleep (N3%) and NfL levels; higher N3% was associated with higher levels of NfL in Non‐Dippers (β = 0.21 [95%CI, 0.06, 0.35, *p* = 0.006), but not in BP Dippers (β = ‐0.006, [95%CI, ‐0.20, 0.19, *p* = 0.952) (Figure 1b). No other significant interactions were identified with any other plasma biomarkers.

**Conclusion:**

In individuals with a non‐dipping BP profile, shorter sleep duration and higher N3% were associated with higher levels of neuronal damage, as measured by plasma NfL levels. The double hit of short sleep and nocturnal hypertension may be detrimental to brain health.